# New Approach for the Calculation of the Intraocular Lens Power Based on the Fictitious Corneal Refractive Index Estimation

**DOI:** 10.1155/2019/2796126

**Published:** 2019-05-14

**Authors:** Joaquín Fernández, Manuel Rodríguez-Vallejo, Javier Martínez, Ana Tauste, David P. Piñero

**Affiliations:** ^1^Department of Ophthalmology (Qvision), Vithas Virgen Del Mar Hospital, 04120 Almería, Spain; ^2^Department of Ophthalmology, Torrecardenas Hospital Complex, 04009 Almería, Spain; ^3^Department of Optics, Pharmacology and Anatomy, University of Alicante, Alicante, Spain; ^4^Department of Ophthalmology, Vithas Medimar International Hospital, Alicante, Spain

## Abstract

**Purpose:**

To identify the sources of error in predictability beyond the effective lens position and to develop two new thick lens equations.

**Methods:**

Retrospective observational case series with 43 eyes. Information related to the actual lens position, corneal radii measured with specular reflection and Scheimpflug-based technologies, and the characteristics of the implanted lenses (radii and thickness) were used for obtaining the fictitious indexes that better predicted the postoperative spherical equivalent (SE) when the real effective lens position (ELP) was known. These fictitious indexes were used to develop two thick lens equations that were compared with the predictability of SRK/T and Barrett Universal II.

**Results:**

The SE relative to the intended target was correlated to the difference between real ELP and the value estimated by SRK/T (ΔELP) (*r* = −0.47, *p*=0.002), but this only predicted 22% of variability in a linear regression model. The fictitious index for the specular reflection (*n*_k_) and Scheimpflug-based devices (*n*_c_) were significantly correlated with axial length. Including both indexes fitted to axial length in the prediction model with the ΔELP increased the *r*-square of the model up to 83% and 39%, respectively. Equations derived from these fictitious indexes reduced the mean SE in comparison to SRK/T and Barrett Universal II.

**Conclusions:**

The predictability with the trifocal IOL evaluated is not explained by an error in the ELP. An adjustment fitting the fictitious index with the axial length improves the predictability without false estimations of the ELP.

## 1. Introduction

Intraocular lens (IOL) power calculation formulas have evolved since the publication of the Fyodorov formula in 1967 [1, 2]. Nowadays, there are several methods for calculating the IOL power that can be classified in one of the following groups: (1) historical/refraction based, (2) regression, (3) vergence, (4) artificial intelligence, and (5) ray tracing [3]. The first two approaches are considered out of date, the artificial intelligence is growing in popularity but not in predictability [4], and the ray tracing [5, 6] is the promising option that has not still replaced the most used methods based on the vergence formula. An important reason for the absence of a clear evidence of differences between these previous three approaches is the inclusion of some regression components in all of them, including ray tracing [3]. In fact, the main difference between vergence formulas is the number of variables used for estimating the effective-thin lens position (ELP_o_), [7] ranging from two in SRK/T, Hoffer Q, and Holladay I formulas to five or seven in the Barrett Universal II or Holladay II formulas, respectively [3].

There are several studies that report the predictability of vergence formulas for eyes with different axial lengths, but high discrepancies are found in the percentage of eyes within ±0.50 D between studies [4, 8–13]. For instance, Shrivastava et al. [14] reported no differences between SRK/T and the newer formulas in short eyes, but a meta-analysis reported superiority of the Haigis formula [15]. The reality is that there are no clinically relevant differences in the statistics of centrality for the postoperative spherical equivalent (SE) between equations, and special attention should be taken in dispersion [10]. This dispersion of the data has been reported to be lower for the Barrett Universal II, which results in a higher percentage of eyes within ±0.50 D in medium to long eyes [4, 8–13]. The Barrett Universal II was born from the theoretical universal formula which considers the thick lens formula [16] and after an estimation of the lens factor, which is the distance from the iris to the second principal plane of the IOL [17]. Therefore, the thin lens formula can be used considering the ELP_o_ as the anterior chamber depth (ACD) plus the lens factor which can be derived from the A-constant [17]. Other authors have used the terms surgeon factor [18] or offset [19] instead of lens factor but the aim of these constants was the same: to estimate the location of the second principle plane of the IOL optic from a relatively fixed anatomical reference plane and to compute the ELP_o_ by means of this factor [16].

If the intended preoperative spherical equivalent (SE) and the postoperative SE are not equal, the constants implemented by different formulas can be optimized for improving refractive results in eyes with different axial lengths [20, 21], but this may contribute to an error in the ELP_o_ if the lens position is not measured during the postoperative follow-up. The aim of this study was to evaluate if the postoperative SE after implantation of a trifocal IOL was due to an error in the ELP_o_ estimated with the SRK/T formula and, if this was not the reason, to identify the possible sources of error. For this purpose, the actual lens position (ALP) of each eye was measured after surgery, and the thick lens formula [22] was used to avoid the optical approximations required by the vergence formula [2].

## 2. Materials and Methods

### 2.1. Subjects and Procedures

The study was approved by the local Ethics Committee and was performed in adherence to the tenets of the Declaration of Helsinki. Data from 43 subjects measured at the 3-month follow-up visit were retrospectively retrieved from our historical database, including only one eye randomly in the analysis. The tomography obtained at this visit with the Pentacam HR (Oculus, Wetzlar, Germany) was used for collecting data including anterior (*r*_1c_) and posterior corneal radii (*r*_2c_), corneal thickness (*e*_c_), and ALP measured from corneal vertex (anterior corneal surface) to the anterior IOL surface. The axial length (AXL), the preoperative ACD, and the anterior corneal radius (*r*_k_) were retrieved from the measurements obtained with the IOLMaster 500 system (Carl Zeiss Meditec AG, Germany). The postoperative best spectacle refraction was also obtained for each eye computing the SE. The pupil diameter for the conditions for which the refraction was performed (around 90 lux) was estimated as the mean between photopic and mesopic pupils measured with the Keratograph 5M system (Oculus, Wetzlar, Germany).

### 2.2. Surgery Procedure

All surgeries were conducted by the same surgeon (X) by means of phacoemulsification or femtosecond laser-assisted cataract surgery (Victus, Bausch & Lomb Inc, Dornach, Germany) through clear corneal incisions of 2.2 mm for manual incisions or 2.5 mm for laser incisions, both at temporal location. The implanted IOL at capsular bag was the Liberty Trifocal (Medicontur Medical Engineering Ltd. Inc., Zsámbék, Hungary) based on the elevated phase shift (EPS) technology, which is an aspheric hydrophilic IOL with +3.50 D of addition for near and +1.75 D for intermediate at the IOL plane. The preoperative calculation of the IOL power was conducted with the SRK/T [19] formula considering the manufacturer recommended constant of 118.9.

### 2.3. Thick Lens Formula

**Figure 1:**
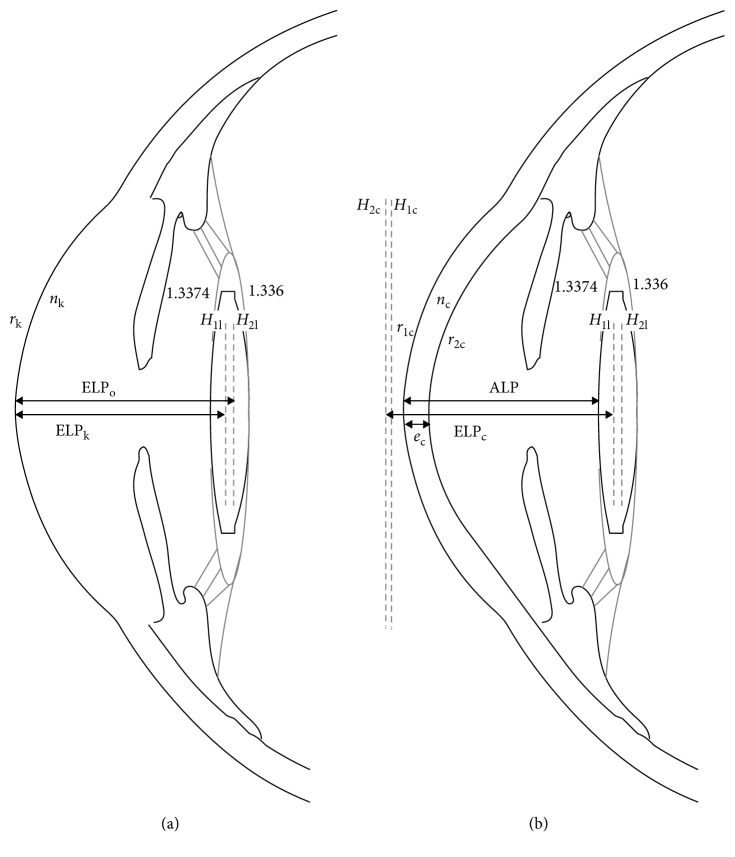
(a) Model for the calculation of the corneal power derived from anterior corneal radius (*r*_k_) and fictitious index (*n*_k_). The effective lens positions for vergence thin lens formula (ELP_o_) and for thick lens formula (ELP_k_) are shown. 1.3374 and 1.336 are the refractive indexes for the aqueous and vitreous, respectively. (b) Schema for computing the IOL power based on the thick lens formula, both anterior (*r*_1c_) and posterior (*r*_2c_) corneal radii were measured, and total corneal power was obtained estimating refractive index of the cornea (*n*_c_). Actual lens position (ALP) and effective lens position (ELP_c_) from principal planes are represented. *H*_1c_, *H*_2c_, H_1l_, and *H*_2l_ are the first and second principal planes for the cornea and the IOL.

All the calculations were conducted by means of paraxial optics and coupling the measured optical structures with the thick lens formula [22]. Some approaches were conducted depending on the system used to measure the cornea. For the anterior corneal radius (*r*_k_) obtained with IOLMaster, the corneal power in equation (1) (*P*_k_) should be estimated with a fictitious index (*n*_k_), and the cornea was considered as a single dioptric surface; therefore, corneal principal planes were approximated to the anterior corneal surface (Figure 1(a)). For the measurement of both corneal radii (*r*_1c_ and *r*_2c_) and corneal thickness (*e*_c_) with the Pentacam (Figure 1(b)), the total corneal power was computed with equation (2), and corneal principal planes were calculated since the cornea was considered as a thick lens (Figure 1(b)) [22]:

(1) $$\begin{array}{c} P_{\text{k}}=\frac{n_{\text{k}}-1}{r_{\text{k}}}, \end{array}$$

(2) $$\begin{array}{c} P_{\text{c}}=\frac{n_{\text{c}}-1}{r_{1\text{c}}}+\frac{1.3374-n_{\text{c}}}{r_{2\text{c}}}-\left(\frac{e_{\text{c}}}{n_{\text{c}}}\right)\left(\frac{n_{\text{c}}-1}{r_{1\text{c}}}\right)\left(\frac{1.3374-n_{\text{c}}}{r_{2\text{c}}}\right). \end{array}$$

The characteristics of the IOL implanted in each patient were provided by the manufacturer, including thickness and anterior and posterior radii (these are not detailed in the results as they were required to be kept as confidential by the manufacturer). Therefore, the principal planes were also calculated for the IOLs (Figure 1). Finally, to calculate the equivalent lens for the coupling of the cornea and the IOL, it was required to define the distances between both optical structures (ELP) taking the principal planes as the reference if possible (Figure 1(b)) or the anterior cornea location when the cornea was considered as a thin lens (Figure 1(a)). Different approximations for the real effective lens position depending on the principal planes location were considered: from corneal vertex to second IOL principal plane (equation (3); Figure 1(a)), from second corneal principal plane to first IOL principal plane (equation (4); Figure 1(b)), and from corneal vertex to first IOL principal plane (equation (5); Figure 1(a)):

(3) $$\begin{array}{c} {\text{ELP}}_{\text{o}}=\text{ALP}+H_{2\text{l}}, \end{array}$$

(4) $$\begin{array}{c} {\text{ELP}}_{\text{c}}=\text{ALP}-H_{2\text{c}}+H_{1\text{l}}, \end{array}$$

(5) $$\begin{array}{c} {\text{ELP}}_{\text{k}}=\text{ALP}+H_{1\text{l}}. \end{array}$$

While equation (3) was the real ELP_o_ that should be predicted for the vergence formula [2], the other two were used in the thick lens formula depending if both corneal surfaces radii (ELP_c_) (equation (4)) or only anterior corneal radius (ELP_k_) (equation (5)) were used.

The SRK/T is a vergence formula; therefore, it predicts what would be the ELP_o_ (equation (3)), considering the biometric eye parameters and an A-constant associated to the IOL. As we measured the postoperative ALP and calculated the principal planes of the IOL, we can calculate the difference between the ELP_o_ estimated preoperatively by the SRK/T formula (hereinafter abbreviated as ELP_SRK/T_) [19] and the real ELP_o_ calculated postoperatively (equation (3)) (ΔELP = ELP_SRK/T_ − ELP_o_). The correlation between ΔELP and the postoperative SE was computed in order to assess the amount of postoperative SE explained by an error in the ELP_o_ estimation by the SRK/T formula.

The predictability obtained with SRK/T and the predictability that would have expected if Barrett Universal II (white to white and lens thickness not considered) [23] had been used were compared with those achievable with the two thick lens equations, for which the corneal power was derived from the measures of the anterior corneal radius measured with the IOLMaster (equation (1); Figure 1(a)) or both corneal surfaces measured with the Pentacam (equation (2); Figure 1(b)). For this purpose, the postoperative SE refraction was adjusted to the intended target refraction computed by each formula for the implanted IOL power [24, 25].

### 2.4. Fictitious Indexes

**Figure 2:**
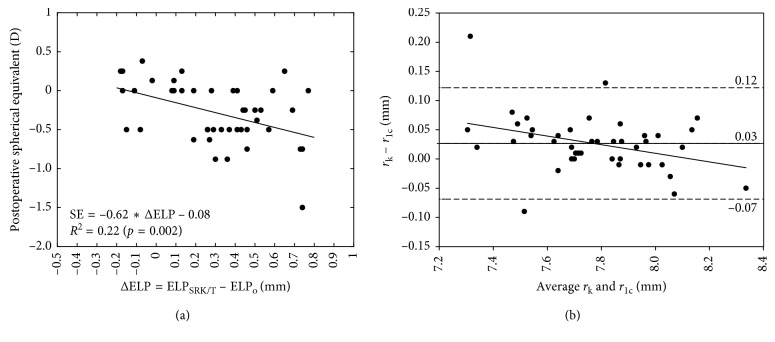
(a) Postoperative spherical equivalent relative to the intended target for the SRK/T versus the difference between the effective lens position estimated by the SRK/T formula (ELP_SRK/T_) and the real obtained from the measurement of the actual lens position and the location of the second principal plane of the IOL (ELP_o_). (b) Agreement between anterior corneal radius measured with IOLMaster (*r*_k_) and Pentacam HR (*r*_1c_).

The fictitious indexes were defined as the refractive indexes used for computing the corneal power that better predicts the postoperative SE after surgery when the real ELP is known. Considering that the corneal radii, ELP, AXL, and the IOL characteristics (radii and thickness) were known after surgery, the only variable for predicting the postoperative SE with the thick lens formula was the fictitious index. Therefore, an iterative process was conducted for obtaining these indexes considering two possibilities: (1) the corneal power was derived from a fictitious index (*n*_c_) considering anterior and posterior corneal radii and corneal thickness (Figure 1(a)) and (2) the corneal power was derived from a fictitious index (*n*_k_) and the anterior corneal radius (Figure 1(b)). This kind of iterative processes has been used for finding the best constant that predicts best the difference between the intended and the actual SE [20]. However, it should be considered that the purpose of refining constants is to correct wrong estimations of ELP_o_. In our study, as the real ELP_o_ was known, other unknown sources of error were investigated and adjusted by modifying the corneal power through the fictitious refractive indexes.

### 2.5. Statistical Analysis

The normality of data distributions for the variables evaluated was tested with the Shapiro–Wilk test, and parametric statistics were selected for testing hypothesis only if the assumptions were met. Correlations were evaluated with the Pearson *r* test, and the paired *t*-test was used for testing differences between real ELP_o_ and ELP_SRK/T_. The thick lens equation [22] and all the functions required to estimate the fictitious indexes by means of iteration processes were implemented in MATLAB (Mathworks, Inc., Natick, MA). The statistical analyses were performed using the IBM SPSS 24.0 software for Windows (SPSS, Chicago, IL). Mean ± standard deviation [median (interquartile range)] is used in Section 3 for reporting central tendency and data dispersion.

## 3. Results

Mean age of the sample was 68 ± 8 [70 (7)] years old. The ΔELP was significantly correlated with the postoperative SE relative to the intended target (*r* = −0.47, *p*=0.002) (Figure 2(a)). A linear regression model predicted that the 22% of the variability in the postoperative SE was explained by an error estimation of the ELP_o_ [*F*(1, 41) = 11.308, *p*=0.002, *R*^2^ = 0.22]. No significant correlations of ΔELP were found with AXL (*r* = 0.23, *p*=0.15) and preoperative ACD (*r* = −0.23, *p*=0.14). The real ELP_o_ was 5.05 ± 0.29 [5.02 (0.32)] mm, and the ELP_SRK/T_ was 5.36 ± 0.31 [5.39 (0.34)] mm (*t* = 7.336, *p* < 0.0005). The anterior corneal radius measured by specular reflection (*r*_k_) overestimated the value measured by the Scheimpflug-based devices in 0.03 ± 0.05 [0.03 (0.05)] mm (*t* = 3.49, *p*=0.001), and the difference was correlated with the average of both measures (*r* = −0.36, *p*=0.02) (Figure 2(b)).

**Figure 3:**
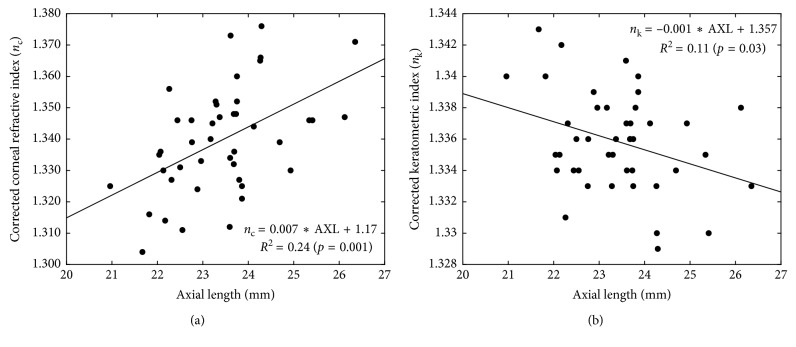
Correlations between the fictitious index (*n*_c_) that minimize the postoperative SE for a known effective lens position considering both corneal radii and thickness (a), and the fictitious index (*n*_k_) derived from the anterior corneal radius (b).

Mean fictitious refractive indexes for *n*_k_ and *n*_c_ were 1.336 ± 0.003 [1.336 (0.004)] and 1.339 ± 0.017 [1.336 (0.021)], respectively. These indexes were correlated with the axial length of the eye, for both *n*_c_ (*r* = 0.49, *p*=0.001) (Figure 3(a)) and *n*_k_ (*r* = −0.33, *p*=0.03) (Figure 3(b)). A multiple linear regression model for predicting postoperative SE relative to the intended target was conducted including ΔELP and *n*_k_ or *n*_c_. The *r*-square increased from 0.22 to 0.83 after including *n*_k_ [*F*(2, 40) = 99.425, *p* < 0.0005, *R*^2^ = 0.83], and from 0.22 to 0.39 after including *n*_c_ [*F*(2, 40) = 12.78, *p* < 0.0005, *R*^2^ = 0.39] in the regression in combination with ΔELP (Table 1).

Two thick equations were developed considering different fictitious indexes depending on axial length. For the thick lens equation considering anterior corneal radius (*n*_k_ equation), *n*_k_ = 1.339 was used for eyes ≤ 22 mm, *n*_k_ = 1.336 for eyes from 22 to 24.5 mm, and *n*_k_ = 1.333 for eyes ≥ 24.5 mm. For the thick lens equation considering anterior and posterior corneal radii, *n*_c_ = 1.328 was used for eyes ≤22 mm, *n*_c_ = 1.339 for eyes from 22 to 24.5 mm, and *n*_c_ = 1.350 for eyes ≥24.5 mm. A multiple linear regression was conducted for predicting ALP considering preoperative ACD and AXL, obtaining the following equation for ALP = 0.527·ACD + 0.102·AXL + 0.41 [*F*(2, 40) = 57.20, *p* < 0.0005, *R*^2^ = 0.74]. The predicted ALP instead of the real measured ALP was used for computing the predictability with both thick lens equations since the ALP should be estimated before surgery.

**Figure 4:**
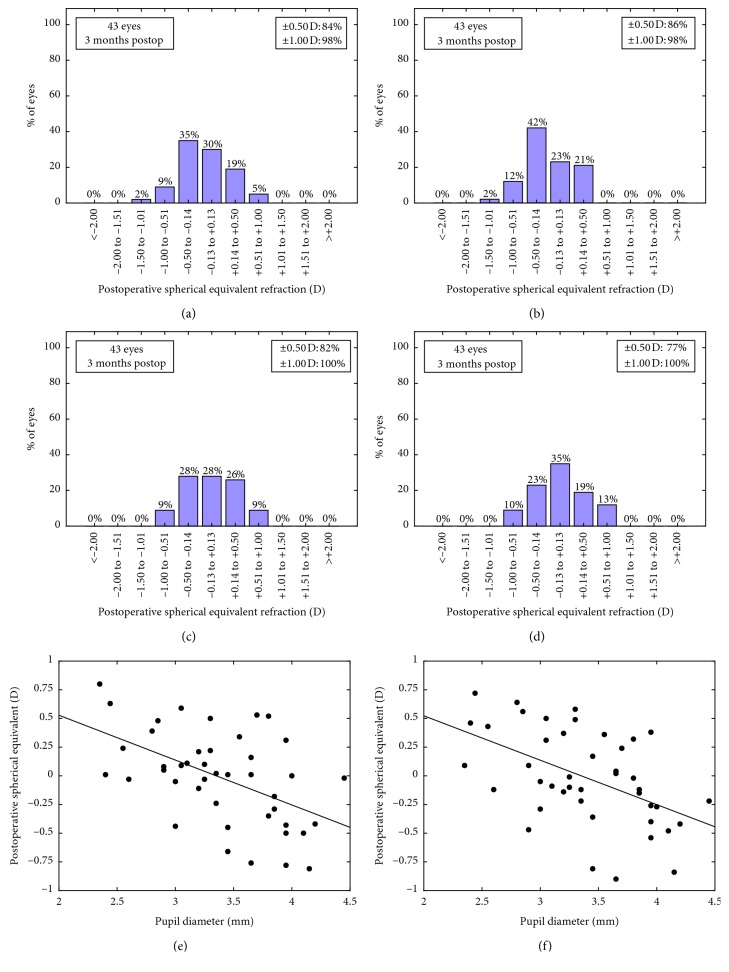
Postoperative spherical equivalent relative to the intended target for (a) SRK/T, (b) Barrett Universal II, (c) *n*_k_ equation, and (d) *n*_c_ equation (*D*). Correlations between the postoperative spherical equivalent relative to the intended target and the pupil diameter for (e) *n*_c_ equation and (f) *n*_k_ equation.

Postoperative SE relative to the intended target was −0.12 ± 0.38 [−0.11 (0.50)] D for the SRK/T (Figure 4(a)), −0.20 ± 0.33 [−0.24 (0.54)] D for Barrett Universal II (Figure 4(b)), −0.01 ± 0.41 [−0.05 (0.62)] D for *n*_k_ equation (Figure 4(c)), and −0.02 ± 0.40 [0.01 (0.57)] D for *n*_c_ equation (Figure 4(d)). The predictability was significantly correlated with pupil diameter for *n*_c_ equation (*r* = −0.50, *p*=0.001) (Figure 4(e)) and for *n*_k_ equation (*r* = −0.51, *p* < 0.0005) (Figure 4(f)). A similar correlation but of less strength was found for Barrett Universal II (*r* = −0.31, *p*=0.04), but not for SRK/T (*r* = −0.04, *p*=0.79).

## 4. Discussion

Optical biometers use the keratometric index (1.3375) for computing the corneal power from anterior corneal radius. However, it is well known that this keratometric index is far from being the one which better predicts the postoperative SE, and current formulas use a fictitious refractive index from 1.3315 to 1.336, close to the tear film refractive index which results in better postoperative SE predictions [7]. The formula used for calculating the IOL power in this study (SRK/T) uses a fictitious index of 1.333, which is within this range. It is well known that SRK/T formula, as any other formulas, reduces the predictability in more or less degree depending on the axial length of the eye, corneal power, and other variables [10]. In our study, we found that an estimated error in the ELP_o_ with the SRK/T formula explained 22% of the variability in the postoperative SE, but a higher percentage of error remained unknown. It is important to note that in the regression of Figure 2(a), we maintained an outlier in the analysis corresponding to the highest postoperative spherical equivalent of −1.50 D. This value can affect the *r*-square value due to the small sample. For this reason, we recomputed the regression equation omitting the outlier, and the *r*-square value decreased to 15% (*p*=0.01). This means that the variability explained by a wrong estimation of the ELP_o_ might be even lower.

Interestingly, we found that when real ELP_o_ is known, the fictitious index can be fitted in order to reduce the predictability error that was not attributed to the ELP_o_. Our mean refractive index for computing corneal power and deriving from anterior corneal surface was *n*_k_ = 1.336 that has been historically reported by Holladay to be close to the tear film [7, 26]. By contrast, *n*_c_ = 1.339 was found when anterior and posterior corneal radii were considered. The latter finding is also quite important because ray tracing and total corneal refractive power have not demonstrated, as theoretically expected, to provide better predictability than current formulas [27–30]. From our results, it can be concluded that a fictitious index is required for computing corneal power derived from anterior corneal radius, whereas a different fictitious index is required for total corneal power calculation.

Fictitious indexes were correlated with AXL, suggesting that fitting these refractive indexes depending on the axial length, the predictability can be increased without overestimating or underestimating the ELP from its real value. The inclusion of *n*_k_ with ΔELP in the model of prediction of postoperative SE relative to the intended target explained 83% of the variability instead of 22%. By contrast, the inclusion of *n*_c_ with ΔELP in the model of prediction of postoperative SE after target correction explained 39% of variability. This means that, even though improving the prediction of the real ELP, there would be some error in the postoperative SE that can be corrected by means of modifying de fictitious index and then the corneal power. Furthermore, even though both approaches can improve the predictability, the corneal power derived from specular reflection devices would lead to better results [31]. Possibly, specular reflection devices compute the radius over the tear film whereas with Scheimpflug devices, tear film is ignored. In fact, we found that the difference in the anterior corneal radius measured with Pentacam HR and IOLMaster systems was correlated with the average from both measures, suggesting that some caution should be considered when using equations derived from measurements of different devices.

A very important consideration is to evaluate the mode of change of these fictitious indexes between different approaches to compute the corneal power. Whereas *n*_k_ decreased with the increase of axial length and *n*_c_ increased in the opposite direction, but with an overestimation of the corneal power with the increase of AXL in both cases. This overestimation can be corrected by means of decreasing *n*_k_ or increasing *n*_c_ with the increment of AXL. Our results are consistent with those reported by Preussner et al. [32] who found an hyperopic outcome in very long eyes (>28 mm), and this was attributed to an overestimation of the corneal power that can be compensated in normal eyes with a systematically overestimated ELP, but not being possible in very long eyes. In fact, even though our sample does not include very long eyes, applying our linear regression for an eye of 30 mm, we obtained the fictitious refractive index (*n*_k_ = 1.327) proposed by Preussner et al. [32] for very long eyes.

The mean postoperative SE relative to the intended target with the thick lens formulas derived from the study was reduced in comparison to SRK/T or Barrett Universal II, both showing a myopic shift that can be explained by the overestimation of ELP_o_ with SRK/T. The higher was the ELP_o_, the lower was the eye power, and consequently, an overestimation of ELP_o_ led to an underestimation of eye power, leading to an overestimating of the required IOL power and resulting in a myopic shift. While the percentage of eyes within ±0.50 D was higher for SRK/T and Barrett Universal II, the percentage of eyes within ±1.00 D was 100% for both thick formulas, with better predictability with *n*_k_ equation in comparison to *n*_c_ equation. The most interesting finding is that the percentage of cases with an error higher than ±0.50 D corresponded to eyes with the highest and lowest pupil diameters, suggesting that a hyperopic shift can be estimated by the formula as a consequence of the presence of small pupils focusing the image in front of the retina, with the opposite trend for large pupils. This suggests that, using these formulas with the trifocal IOL evaluated in our series, a trend to plus target should be used in patients with smaller pupils and to a minus target in eyes with larger pupils as choosing a negative target in small pupils can lead to higher myopic residuals than those predicted by the formula and vice versa. This reasoning can be also valid for Barrett Universal II after constant fitting, but not for SRK/T for which the correlation with pupil diameter was not significantly manifested.

This study is a first approach for the development of new thick lens equations that can be used when measuring the corneal geometry with specular reflection or Scheimpflug-based devices, but it has some limitations that should be considered. First, the small sample for a particular surgeon supposes that the ALP prediction formula might not be transferable to other surgeons. Higher samples with results obtained from different surgeons are required for a general estimation of the ALP. Second, the sample included eyes with AXL ranging from 20.96 to 26.35 mm and therefore with low number of short and long eyes in comparison to medium or medium-long eyes. Although the tendency of *n*_k_ and *n*_c_ is clear with axial length, an improvement in the estimation of the fictitious indexes would be obtained by increasing the number of eyes, especially for short, long, and very long eyes. Finally, the predictability of these new formulas has been computed in the same sample for which they were developed. The performance of new studies with these formulas in a different sample for confirming the results of predictability is needed. In fact, in our opinion, future crossover studies are required assigning different formulas to uniform groups instead of predicting what would have happened if different formulas had been used as the current comparative studies of formulas are doing.

In conclusion, in this study, we have demonstrated that the postoperative SE with some of the current vergence formulas can be due to the result of an underestimation or overestimation of the real ELP. However, even if the real ELP was perfectly predicted before surgery, some postoperative SE can appear depending on axial length. This could be corrected by means of fitting the constant of the formula leading to a false ELP prediction or by means of optimizing the fictitious indexes for different axial lengths. We have also demonstrated that the second option reduces the mean postoperative SE, either for specular reflection devices or Scheimpflug-based devices. However, it is important to consider that the slopes of correlation between both approaches are of opposite sign. Another very interesting finding is that higher errors of predictability can be due to pupil diameter changes during refraction with the used multifocal intraocular lens. We suggest to include the pupil diameter in predictability studies for exploring this finding with other multifocal or monofocal IOLs.

## Figures and Tables

**Table 1:** Multiple regression linear models for prediction of postoperative SE relative to the intended target.

Variable	*B*	SEB	*β*	*t*	*p*
Intercept	154.55	12.73		12.14	<0.0005
ΔELP (mm)	−1.44	0.11	−1.07	−13.10	<0.0005
*n*_k_	−115.45	9.52	−0.99	−12.13	<0.0005

Intercept	−14.10	4.20			0.002
ΔELP (mm)	−0.98	0.20	−0.73	−4.99	<0.0005
*n*_c_	10.67	3.16	0.50	3.38	0.002

## Data Availability

The data supporting the results of the current article are available from the corresponding author upon request.
